# Subcellular Localization of Rice Acyl-CoA-Binding Proteins ACBP4 and ACBP5 Supports Their Non-redundant Roles in Lipid Metabolism

**DOI:** 10.3389/fpls.2020.00331

**Published:** 2020-03-24

**Authors:** Pan Liao, King Pong Leung, Shiu-Cheung Lung, Saritha Panthapulakkal Narayanan, Liwen Jiang, Mee-Len Chye

**Affiliations:** ^1^School of Biological Sciences, The University of Hong Kong, Pokfulam, China; ^2^State Key Laboratory of Agrobiotechnology, CUHK, New Territories, China; ^3^Centre for Cell and Development Biology and State Key Laboratory of Agrobiotechnology, School of Life Sciences, The Chinese University of Hong Kong, New Territories, China

**Keywords:** acyl-CoA-binding protein, *Oryzae sativa*, pathogen treatment, salt treatment, subcellular localization

## Abstract

Acyl-CoA-binding proteins (ACBPs), conserved at the acyl-CoA-binding domain, can bind acyl-CoA esters as well as transport them intracellularly. Six ACBPs co-exist in each model plant, dicot *Arabidopsis thaliana* (thale cress) and monocot *Oryza sativa* (rice). Although Arabidopsis ACBPs have been studied extensively, less is known about the rice ACBPs. *OsACBP4* is highly induced by salt treatment, but down-regulated following pathogen infection, while *OsACBP5* is up-regulated by both wounding and pathogen treatment. Their differential expression patterns under various stress treatments suggest that they may possess non-redundant functions. When expressed from the *CaMV35S* promoter, OsACBP4 and OsACBP5 were subcellularly localized to different endoplasmic reticulum (ER) domains in transgenic Arabidopsis. As these plants were not stress-treated, it remains to be determined if OsACBP subcellular localization would change following treatment. Given that the subcellular localization of proteins may not be reliable if not expressed in the native plant, this study addresses *OsACBP4:GFP* and *OsACBP5:DsRED* expression from their native promoters to verify their subcellular localization in transgenic rice. The results indicated that OsACBP4:GFP was targeted to the plasma membrane besides the ER, while OsACBP5:DsRED was localized at the apoplast, in contrast to their only localization at the ER in transgenic Arabidopsis. Differences in tagged-protein localization in transgenic Arabidopsis and rice imply that protein subcellular localization studies are best investigated in the native plant. Likely, initial targeting to the ER in a non-native plant could not be followed up properly to the final destination(s) unless it occurred in the native plant. Also, monocot (rice) protein targeting may not be optimally processed in a transgenic dicot (Arabidopsis), perhaps arising from the different processing systems for routing between them. Furthermore, changes in the subcellular localization of OsACBP4:GFP and OsACBP5:DsRED were not detectable following salt and pathogen treatment, respectively. These results suggest that OsACBP4 is likely involved in the intracellular shuttling of acyl-CoA esters and/or other lipids between the plasma membrane and the ER, while OsACBP5 appears to participate in the extracellular transport of acyl-CoA esters and/or other lipids, suggesting that they are non-redundant proteins in lipid trafficking.

## Introduction

During lipid biosynthesis and metabolism, lipids including acyl-CoA esters and their derivatives are transferred within and across subcellular compartments (Xiao and Chye, 2011a). The ATP-binding cassette (ABC) transporter proteins, lipid-transfer proteins (LTPs) and acyl-CoA-binding proteins (ACBPs) are candidates for facilitating lipid transfer in plants (Rea, 2007; Kang et al., 2011; Xiao and Chye, 2011a; Yurchenko et al., 2014). The ABC transporter proteins have been reported to participate in the transport of fatty acids, acyl-CoA esters, and wax/cutin components (Rea, 2007; Benning, 2009; Kang et al., 2011; Hurlock et al., 2014; Block and Jouhet, 2015; Fan et al., 2015; Hwang et al., 2016; Li-Beisson and Nakamura, 2016; Lefèvre and Boutry, 2018) while the LTPs have been reported to be involved in phospholipid and sulfolipid transfer between organelles (Kader et al., 1996; Kader, 1997; Jouhet et al., 2007; Liu et al., 2015). Previous reports on yeast and mammalian 10 kDa ACBPs have demonstrated their role in the trafficking of acyl-CoA esters within the cell, in maintaining an acyl-CoA pool and in glycerolipid biosynthesis (Schjerling et al., 1996; Knudsen et al., 2000; Faergeman and Knudsen, 2002). Our investigations on the six Arabidopsis ACBPs (AtACBPs) indicated their functions in maintaining an acyl-CoA pool and in glycerolipid biosynthesis, affecting plant development, stress responses, membrane biogenesis and signaling pathways (Xiao and Chye, 2011a; Du et al., 2016; Lung and Chye, 2016a, 2019; Lung et al., 2017, 2018; Chen et al., 2018; Hu et al., 2018). Furthermore, participation of AtACBPs in lipid trafficking based on their different subcellular localization has been proposed (Xiao and Chye, 2011a; Lung and Chye, 2016b). AtACBP2 and AtACBP3 were found to be non-redundant in Arabidopsis (Chye et al., 2000; Li and Chye, 2003; Chen et al., 2010; Gao et al., 2010) because AtACBP2 was localized at the plasma membrane (PM) and endoplasmic reticulum (ER) (Li and Chye, 2003) while AtACBP3 was targeted to the apoplast and is known to be a phloem-mobile protein (Zheng et al., 2012; Leung et al., 2006; Hu et al., 2018). Recombinant AtACBP2 has been observed to bind 18:1 phosphatidylcholine (PC) and 18:2 PC, lyso PC and several acyl-CoA esters (18:2 > 18:3 > 20:4∼16:0 > 18:1) *in vitro* (Chye et al., 2000; Chen et al., 2010; Gao et al., 2010). Together with AtACBP1, AtACBP2 was reported to play an important role in early embryogenesis, seed germination and seedling development (Chen et al., 2010; Du et al., 2013b). Furthermore, the overexpression of AtACBP2 protected transgenic Arabidopsis from drought stress (Du et al., 2013a). AtACBP2 is known to interact with its protein partners, LYSOPHOSPHOLIPASE2 (LYSOPL2) and a heavy-metal-binding farnesylated protein (FP6), via its ankyrin repeats and transgenic Arabidopsis overexpressing AtACBP2, AtLYSOPL2, or AtFP6 were more tolerant to oxidative (H_2_O_2_)- and cadmium-induced stress (Gao et al., 2009, 2010; Miao et al., 2019).

Recombinant AtACBP3 binds phospholipids PC and phosphatidylethanolamine (PE), polyunsaturated 20:4-CoA and unsaturated C18-CoA esters *in vitro* (Leung et al., 2006; Xiao et al., 2010; Hu et al., 2018). The overexpression of AtACBP3 accelerated starvation-induced and age-dependent leaf senescence (Xiao et al., 2010). Also, AtACBP3 was found to regulate autophagy-mediated leaf senescence by affecting the formation of ATG8-PE through interaction with PE (Xiao and Chye, 2010; Xiao et al., 2010). Transgenic Arabidopsis overexpressing AtACBP3 were upregulated in the expression of pathogenesis-related (PR) genes, displayed induced cell death that resulted in H_2_O_2_ and salicylic acid (SA) accumulation, and showed enhanced NON-EXPRESSOR OF PR GENES1 (NPR1)-dependent plant resistance to *Pseudomonas syringae* pv *tomato DC3000* (Xiao and Chye, 2011b), indicating a role of AtACBP3 in SA-dependent plant defense signaling. Microarray data on AtACBP3-overexpressors further confirmed AtACBP3 function in plant defense because many biotic and abiotic stress-related genes including *PR1*, *PR2*, and *PR5* were upregulated (Xiao and Chye, 2011b). More recently, AtACBP3 was reported to be involved in response to hypoxia by regulating very-long-chain fatty acid metabolism (Xie et al., 2015). The enhanced hypoxic tolerance in AtACBP3-overexpressors was dependent on NPR1 and CONSTITUTIVE TRIPLE RESPONSE1 (CTR1)-associated signaling pathways (Xie et al., 2015).

In the monocot *Oryza sativa* (rice), six ACBPs co-exist as in Arabidopsis (Meng et al., 2011). OsACBP1 and OsACBP2 were localized to the cytosol, while OsACBP3 was located in the cytosol and membranous structures (Meng et al., 2014). OsACBP4 and OsACBP5 were targeted to the ER (Meng et al., 2014; Meng and Chye, 2014), and OsACBP6 to the peroxisome (Meng et al., 2014). Although both GFP-tagged OsACBP4 and OsACBP5 were detected at ER-derived spherical structures and membranes of ER bodies in transgenic *OsACBP4:GFP* and *OsACBP5:GFP* Arabidopsis in which the reported fusions were driven by the *CaMV35S* promoter, only OsACBP4:GFP was observed in the central ER cisternae (Meng and Chye, 2014). Recombinant OsACBP4 was reported to bind PA (16:0, 18:0, 18:1), PC (18:0, 18:1, 18:2) and acyl-CoA esters (18:2 > 16:0 > 18:3) (Meng et al., 2011; Meng, 2012). Recombinant OsACBP5 was also shown to bind PA (18:0, 18:1), PC (18:0, 18:1, 18:2) and acyl-CoA esters (18:3 > 16:0) (Meng et al., 2011; Meng, 2012).

Upon abiotic stress treatment including salt and drought, the unfolded protein response (UPR) or NPR1 signaling pathway is triggered to protect cells (Wang et al., 2005; Srivastava et al., 2013, and references cited therein), and lipid compositional changes occur rapidly and dynamically as a consequence (Wang, 2004; Chen et al., 2008; Du Z.-Y. et al., 2010; Xiao and Chye, 2011b; Liao et al., 2014a). While *OsACBP4* was highly induced by salt treatment, it was down-regulated following pathogen infection (Meng et al., 2011). In contrast, the expression of *OsACBP5* was up-regulated by both wounding and pathogen treatment (Meng et al., 2011). The differential subcellular expression patterns of *OsACBP4* and *OsACBP5* under these various stress treatments suggest that they may possess different functions. Although the subcellular localization of OsACBP4 and OsACBP5 had been previously investigated in transgenic *35S:OsACBP4:GFP* and *35S:OsACBP5:GFP* Arabidopsis, the plants were not subjected to any stress treatment (Meng et al., 2014; Meng and Chye, 2014). Interestingly OsACBP4 and OsACBP5 are classified into different ACBP classes consistent with their proposed variation in subcellular function (Meng et al., 2011). OsACBP4, the homolog of AtACBP2, is in class II, while OsACBP5, the homolog of AtACBP3, belongs to class III (Meng et al., 2011; Du et al., 2016). Given that the subcellular localization of proteins may not be reliable if they are not expressed in the native plant (Tian et al., 2004; Zhang et al., 2011; Bu et al., 2014), further investigations were carried out herein to study the subcellular localization of OsACBP4 and OsACBP5 using their native promoters in *OsACBP4promoter:OsACBP4:GFP* and *OsACBP5promoter:OsACBP5:DsRED* fusions expressed in stably-transformed rice. In this study, we demonstrated that OsACBP4:GFP was targeted to the PM besides the ER, while OsACBP5 was localized at the apoplast. Furthermore, changes in the subcellular localization of OsACBP4:GFP and OsACBP5:DsRED were not obvious under salt and pathogen treatment, respectively. These results suggest that OsACBP4 is involved in the intracellular transport of acyl-CoA esters and/or other lipids between the plasma membrane and the ER, while OsACBP5 appears to participate in the extracellular transport of acyl-CoA esters and/or other lipids. This study suggests that OsACBP4 and OsACBP5 are non-redundant proteins in lipid metabolism and provides clues on their functional localization in rice, a crop plant.

## Materials and Methods

### Plant Material and Growth Conditions

*Japonica* rice (*Oryza sativa* cv Zhonghua11) was used in this study. Seeds of wild-type (WT) and transgenic rice were surface-sterilized with 75% ethanol for 1 min, soaked in 25% sodium hypochlorite for 20 min, rinsed four times in sterilized water, and germinated on half-strength Murashige and Skoog (1/2MS) medium, with and without 50 μg ml^–1^ hygromycin. Rice seeds and seedlings were grown in magenta GA-7 plant tissue culture boxes at 25°C (16 h light)/22°C (8 h dark).

### Stress Treatments

Four-, seven-, and fourteen-day-old seedlings were subjected to abiotic or biotic stress treatments. Salt treatment was performed as previously described (Meng et al., 2011) with a minor modification. Briefly, to test the expression of *OsACBP4* after salt treatment, the roots of 7- and 14-day-old WT seedlings were submerged in 200 mM sodium chloride (NaCl) solution, or distilled water as controls, and WT root samples were collected at 0, 6, 12, 24, and 48 h after treatment for RNA extraction (Islam et al., 2009). To test the subcellular localization of OsACBP4 after salt treatment, the roots of 4-day-old seedlings of three independent transgenic *OsACBP4promoter:OsACBP4:GFP*-DX2181 lines (876-5, 876-7, and 876-11) were similarly treated for 24 h before confocal laser-scanning microscopy.

For pathogen treatment, *Rhizoctonia solani* AG-1-1 (ATCC 66157), which causes sheath blight in rice, was used. *R. solani* was inoculated as previously reported (Panthapulakkal Narayanan et al., 2019). To test *OsACBP5* expression after pathogen treatment, 7- and 14-day-old WT seedlings were transferred to fresh Petri dishes containing filter paper wetted with 1 ml of sterilized water. Subsequently, infection was performed by inoculating *R. solani* agar plugs on roots. WT root samples were collected at 0, 6, 12, 18, 24, and 48 h after treatment for RNA extraction. To test the subcellular localization of OsACBP5 after pathogen treatment, 4-day-old rice seedlings of three independent transgenic *OsACBP5promoter:OsACBP5:DsRED* lines (927-9, 927-19, and 927-26) were transferred to fresh Petri dishes and infected for 18 h, using rice roots without infection as controls. Rice roots 18 h post-inoculation (hpi) were used in confocal laser-scanning microscopy. All these experiments were performed independently three times. For the plasmolysis experiments, the roots of 4-day-old seedlings were treated with 0.8 M mannitol for 2 h with shaking (Zhang et al., 2012).

### Construction of Plasmids and Rice Transformation

The *OsACBP4promoter*-DX2181 construct was generated by inserting a 1.2 kb *Sal*I-*Hin*dIII 5′-flanking region of *OsACBP4* amplified by primers ML2465/ML2823 from rice Zhonghua11 (ZH11), into pGEM-T Easy vector (Promega) and cloning this 1.2 kb *OsACBP4pro* fragment into their corresponding sites in binary vector DX2181 (Du H. et al., 2010) to obtain plasmid pOS917. Then a 1.71 kb *Hin*dIII-*Bst*EII fragment of *OsACBP4* coding sequence (CDS) (without its stop codon) fused with GFP was amplified from pOS871 using ML2824/ML2825, inserted into pGEM-T Easy vector (Promega) and cloned into their corresponding sites in pOS917 to obtain plasmid pOS876 (*OsACBP4promoter:OsACBP4:GFP*-DX2181).

To obtain a construct of *OsACBP5promoter:OsACBP5: DsRED*-DX2181, a 2.2 kb *Hin*dIII-*Bam*HI fragment of the *OsACBP5* promoter was amplified by primers ML2470/ML2473 from rice ZH11, inserted into pGEM-T Easy vector (Promega), and cloned into the same sites of the DX2181 vector to form construct pOS820. The 1.71 kb *Bam*HI-*Xba*I fragment of the *OsACBP5 CDS* without its stop codon was amplified using ML2982/ML2983 from pOS581 (Meng et al., 2014) and cloned into pGEM-T Easy vector (Promega) to generate construct pOS924. The 0.69 kb *Xba*I-*Sac*I fragment of *DsRED* was amplified from pGDR plasmid using ML2984/ML2985 and cloned into pGEM-T Easy vector (Promega) and cloned into the same sites of pOS924 to generate construct pOS926. Finally, the 2.4 kb *Bam*HI-*Sac*I fragment of the *OsACBP5* CDS without its stop codon fused with DsRED was excised from vector pOS926 and cloned into the same sites of vector pOS820 to form construct pOS927 (*OsACBP5promoter:OsACBP5:DsRED*-DX2181). DNA sequence analysis was performed to confirm the PCR fragments and inserts in all intermediate and final constructs.

### Generation and Molecular Analyses of Transgenic Rice Lines

The final pOS876 and pOS927 constructs were sent to BioRun^1^ (Wuhan, China) for rice ZH11 transformation as described previously (Guo et al., 2019). The transformed T_0_ seeds were screened on MS medium containing 50 μg ml^–1^ hygromycin and the resistant transformants were further verified by polymerase chain reaction (PCR) (Supplementary Figures S1A, S3A), quantitative reverse transcription PCR (qRT-PCR) (Supplementary Figures S1B, S3B) and western blot analysis (Supplementary Figures S1C, S3C) using rabbit polyclonal anti-GFP or anti-Red fluorescent proteins (RFP) antibodies. Putative transgenic rice *OsACBP4promoter:OsACBP4:GFP*-DX2181 and *OsACBP5promoter:OsACBP5:DsRED*-DX2181 lines were designated as lines 876 and 927, respectively. Finally, three lines with relatively high expression of OsACBP4 (876-5, 876-7 and 876-11) or OsACBP5 (927-9, 927-19 and 927-26), respectively, in mRNA and protein levels, as well as enough seeds were selected from each construct for subsequent analyses.

### Polymerase Chain Reaction (PCR)

To screen transgenic rice *OsACBP4promoter:OsACBP4:GFP*-DX2181 and *OsACBP5promoter:OsACBP5:DsRED*-DX2181 lines, PCR was conducted using a gene-specific forward primer in the *OsACBP4* CDS (ML1060) with a reverse primer in *GFP* (ML2825), or a gene-specific forward primer in *OsACBP5* CDS (ML1111) with a reverse primer in DsRED (ML2985), respectively. The conditions for PCR were as follows: 94°C held for 5 min, followed by 35 cycles of 94°C for 30 S, 57°C for 30 S, and 72°C for 1 min, and a final extension at 72°C for 10 min.

### Quantitative Reverse Transcription PCR (qRT-PCR)

Total RNA from 1-, 2-, and 3-week-old old rice seedlings grown under normal condition, and after salt or pathogen treatments, were extracted with the RNeasy Plant Mini Kit (Qiagen, Hilden, Germany). The RNA (2 μg) was treated by DNase I (Qiagen) before reverse-transcription to first-strand cDNA using the EvoScript Universal cDNA Master Kit (Roche, Mannheim, Germany). Quantitative reverse transcription PCR (qRT-PCR) was performed with FastStart Universal SYBR Green Master Kit (Roche, Mannheim, Germany) on a StepOne Plus Real-time PCR System (Applied Biosystems, Foster City, CA, United States). The conditions for qRT-PCR were as follows: denaturation at 95°C for 10 min, followed by 40 cycles of 95°C for 15 s and 60°C for 30 s (Liao et al., 2019). Three experimental replicates for each reaction were carried out using *OsACBP4* or *OsACBP5* gene-specific primers (ML1109/ML1110 for *OsACBP4* and ML1111/ML1112 for *OsACBP5*), and the rice *ACTIN* (ML1115/ML1116) was used as an internal control. The qRT-PCR data were analyzed by the 2^–ΔΔCt^ method (Schmittgen and Livak, 2008). The relative expression was normalized to *OsACTIN*, and the relative mRNA levels in each transgenic rice line and the empty vector control were analyzed using data from three independent experiments. Significant differences between various samples were analyzed by the Student’s *t*-test. Primers for qRT-PCR are shown in Supplementary Table S1.

### Western Blot Analysis

Rice total protein was extracted (Chye et al., 1999; Liao et al., 2014b) from 1-week-old fresh rice leaves and protein concentration was measured by the Bradford assay (Bradford, 1976). Proteins (20 μg) were resolved on 10% SDS-PAGE and transferred to polyvinylidene difluoride membranes (Pall). Western blot analysis was performed as described previously (Lung et al., 2017; Liao et al., 2018). The blots were cross-reacted with rabbit polyclonal anti-GFP (1: 5000, A6455; Invitrogen) for *OsACBP4promoter:OsACBP4:GFP* transgenic lines or rabbit polyclonal anti-RFP (1:7000, R10367; Invitrogen) for *OsACBP5promoter:OsACBP5:DsRED* transgenic lines at 4°C overnight, followed by incubation with horseradish peroxidase-conjugated anti-rabbit secondary antibodies (1:50,000; Sigma-Aldrich) at room temperature for 1 h. Cross-reacting bands were detected by the Amersham ECL Prime Detection Reagent (GE Healthcare).

### Confocal Laser-Scanning Microscopy

Four-day-old transgenic rice roots under different treatments were examined with a Zeiss LSM710 NLO confocal laser scanning microscope with a 63 × oil lens following Lung et al. (2017) or a Leica SP8 confocal microscope equipped with a 63 × water lens. Fluorescent signals were detected using the following excitation and emission wavelengths: GFP (488 nm/495–545 nm), DsRED (552 nm/560–660 nm), ER-Tracker (594 nm/615 nm), FM 4–64 (488 nm/560–660 nm), 4’,6-diamidino-2-phenylindole (DAPI) (405 nm/410–480 nm). Images were processed using ZEN or LAS X software. For colocalization with an ER marker, 4-day-old seedlings were vacuum infiltrated with the ER-Tracker Red dye (1 μM, Invitrogen, E34250) for 30 min before two 5 min washes in distilled, deionized water (Lung et al., 2018). For colocalization with a PM marker, 4-day-old seedlings were incubated with the FM 4–64 dye (6 μM, Invitrogen, T13320) for 2 min. For colocalization with DAPI, 4-day-old seedlings were vacuum infiltrated with the DAPI dye (10 μg ml^–1^, Roche, 10236276001) for 15 min (Schoor et al., 2015).

### Statistical Analysis

Significant differences in gene expression of *OsACBP4* or *OsACBP5* between different samples were analyzed by the Student’s *t*-test.

## Results

### *OsACBP4* and *OsACBP5* Are Stress Responsive in Rice Roots

**FIGURE 1:**
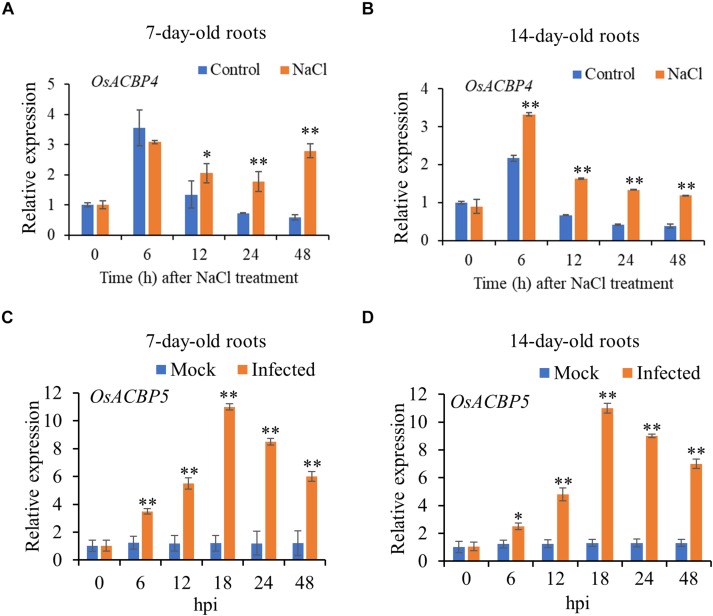
Expression of *OsACBP4* and *OsACBP5* after stress treatment in wild-type (WT) rice roots. **(A)** *OsACBP4* expression in 7-day-old NaCl-treated roots; **(B)** *OsACBP4* expression in 14-day-old NaCl-treated roots; **(C)** *OsACBP5* expression in 7-day-old *Rhizoctonia solani*-treated roots; **(D)** *OsACBP5* expression in 14-day-old *R. solani*-treated roots. Total RNA was extracted from 7- and 14-day-old rice after NaCl treatment or after *R. solani* treatment at 0, 6, 12, 18, 24, and 48 h. Data are means ± SD of three independent replicates. Asterisks indicate a significant difference between the treatments and controls (*P < 0.05; **P < 0.01 by Student’s *t*-test). hpi, hours post infection.

Results from qRT-PCR on 7- and 14-day-old rice roots indicated that *OsACBP4* was induced by salt treatment and *OsACBP5* activated by *R. solani* (Figures 1A–D). In 7-day-old roots, *OsACBP4* was induced at 12 h after salt treatment and its expression remained at relatively high levels from 24 h to 48 h (Figure 1A). In 14-day-old roots, *OsACBP4* peaked at 6 h and its expression maintained at relatively high levels from 12 to 48 h (Figure 1B). *OsACBP5* expression gradually increased from 6 to 18 h after *R. solani* treatment and then decreased, but was retained above their corresponding controls from 24 to 48 h in 7- and 14-day-old roots (Figures 1C,D). These results on salt and pathogen (*R. solani*) induction of expression led us to test the subcellular localization of OsACBP4 and OsACBP5 in young rice roots.

### Localization of OsACBP4:GFP in the Plasma Membrane (PM) and ER in Rice Roots

**FIGURE 2:**
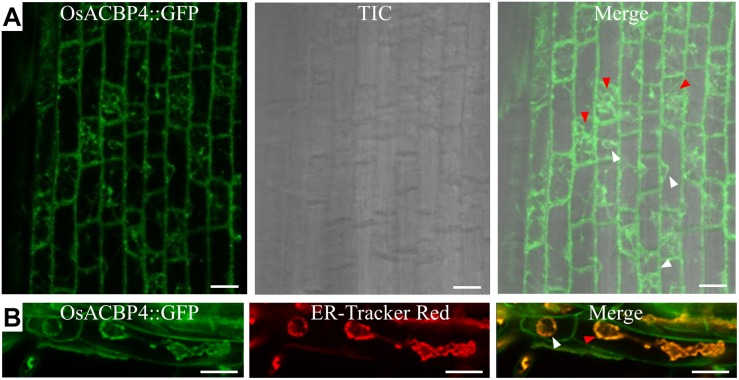
Subcellular localization of OsACBP4:GFP in transgenic rice. **(A)** Root cells of 4-day-old transgenic rice seedlings were imaged by confocal laser scanning microscopy. Signals were detected at the central cisternal ER (red arrow), the perinuclear ER (white arrow) and plasma membrane or cell wall. **(B)** Colocalization of OsACBP4:GFP (green) with ER-Tracker Red (red) (E34250, Invitrogen) in root cells of 4-day-old transgenic rice seedlings. Signals were colocalized at the central cisternal ER (red arrows), the perinuclear ER (white arrows) and plasma membrane or cell wall. Bars = 20 μm. ACBP, acyl-CoA-binding protein; ER, endoplasmic reticulum; Os, *Oryza sativa*. Representative images were shown after observation with consistent results from at least 20 cells in **(A)** for central cisternal ER and perinuclear ER and in **(B)** for cisternal ER. Representative images were shown after observation with consistent results from at least 6 cells for perinuclear ER in **(B)**.

**FIGURE 3:**
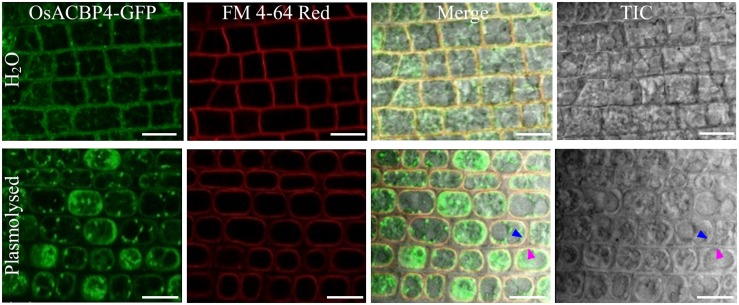
Subcellular localization of OsACBP4:GFP in transgenic rice after plasmolysis. Root cells of 4-day-old transgenic rice seedlings were imaged by confocal laser scanning microscopy. Colocalization of OsACBP4:GFP (green) with FM 4–64 Red (red) (T13320, Invitrogen) in root cells of 4-day-old transgenic rice seedlings. Blue arrow indicates the plasma membrane and pink arrow shows the cell wall. Signals were colocalized at the plasma membrane but not the cell wall. Bars = 20 μm. ACBP, acyl-CoA-binding protein; Os, *Oryza sativa*. Representative images were shown after observation with consistent results from at least 20 cells.

The presence of the *OsACBP4:GFP* transgene in rice was verified by PCR (Supplementary Figure S1A), qRT-PCR (Supplementary Figure S1B), and western blot analyses (Supplementary Figure S1C). When the localization of OsACBP4:GFP was investigated in 4-day-old *OsACBP4promoter:OsACBP4:GFP* transgenic rice roots, the results indicated that OsACBP4:GFP signals were localized at the central cisternal ER (red arrows), the perinuclear ER (white arrows) and the PM or cell wall (Figure 2A). The ER localization of OsACBP4:GFP was further confirmed by its colocalization with the ER marker (Figure 2B). When OsACBP4 was further checked for its localization at the PM or cell wall by using 4-day-old *OsACBP4promoter:OsACBP4:GFP* transgenic rice seedlings subjected to cell plasmolysis under mannitol treatment, the results showed that the OsACBP4:GFP (green) signals colocalized with the membrane marker FM 4-64 (red) at the PM in rice root cells (Figure 3). These results demonstrated that OsACBP4 not only localized at the ER, but also at the PM. However, no changes in the localization of OsACBP4 were noted after salt treatment in rice roots (Supplementary Figure S2).

### Localization of OsACBP5:DsRED in the Apoplast in Rice Roots

**FIGURE 4:**
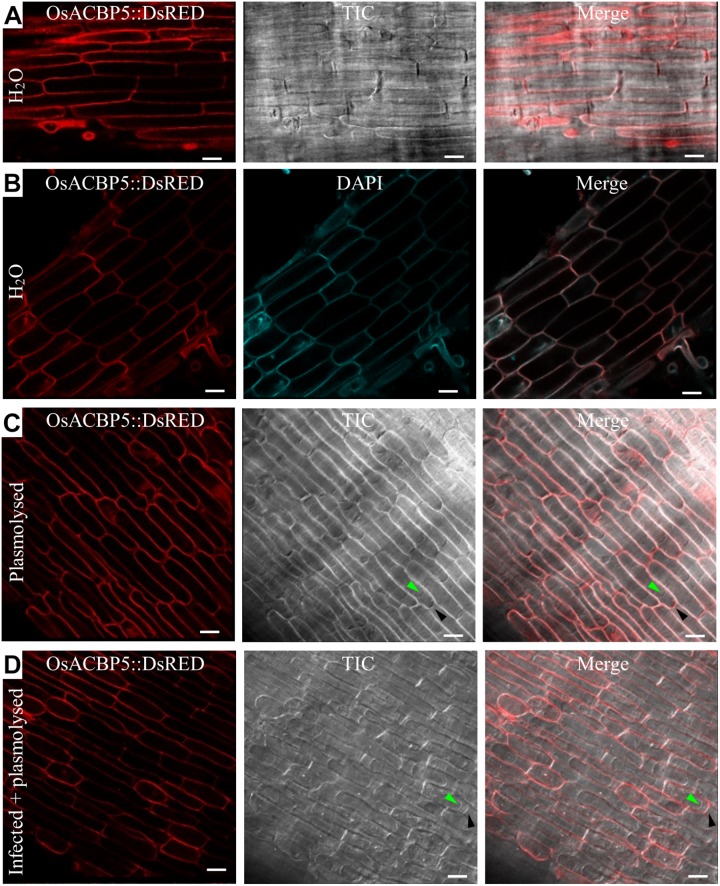
Subcellular localization of OsACBP5:DsRED in transgenic rice after plasmolysis and *Rhizoctonia solani* infection. Root cells of 4-day-old transgenic rice seedlings were imaged by confocal laser scanning microscopy. **(A)** Signals of OsACBP5:DsRED were detected at plasma membrane or cell wall when incubated in water. **(B)** Colocalization of OsACBP5:DsRED (red) with 4′,6-diamidine-2′-phenylindole dihydrochloride (DAPI) (blue) (10236276001, Roche) at cell wall in root cells of 4-day-old transgenic rice seedlings when incubated in water. As DAPI cannot penetrate the live cell, it was used as a cell wall/extracellular space stain here. **(C)** Signals of OsACBP5:DsRED were detected at cell wall in root cells of 4-day-old transgenic rice seedlings after plasmolysis. **(D)** Signals of OsACBP5:DsRED were detected at cell wall in root cells of 4-day-old transgenic rice seedlings after infection and plasmolysis. Green arrow indicates plasma membrane and black arrow shows cell wall. Bars = 20 μm. ACBP, acyl-CoA-binding protein; Os, *Oryza sativa*. Representative images are presented following observation with consistent results from at least 20 cells.

The presence of the *OsACBP5:DsRED* transgene in rice was verified by PCR (Supplementary Figure S3A), qRT-PCR (Supplementary Figure S3B), and western blot analyses (Supplementary Figure S3C). When the localization of OsACBP5:DsRED was investigated in 4-day-old *OsACBP5promoter:OsACBP5:DsRED* transgenic rice roots, the results showed that signals were localized in either the PM or the apoplast (Figure 4A). Given that DAPI cannot penetrate live cells effectively, it was used as cell wall/extracellular space stain, the results indicated that OsACBP5:DsRED signals colocalized with DAPI in rice roots, suggesting that OsACBP5 is localized at the cell wall or extracellular space (Figure 4B). When the localization of OsACBP5 was further addressed by using 4-day-old *OsACBP5promoter:OsACBP5:DsRED* transgenic rice seedlings under mannitol treatment to induce plasmolysis, the results showed that OsACBP5:DsRED signals were maintained at the apoplast in rice root cells (Figure 4C). However, no differences in localization of OsACBP5 were noted after pathogen treatment and plasmolysis in rice roots (Figure 4D).

## Discussion

### OsACBP4- and OsACBP5-Tagged Proteins Are Localized Differentially in Transgenic Rice Roots

In contrast to previous observations in transgenic Arabidopsis root cells that OsACBP4 and OsACBP5, when regulated from the *CaMV35S* promoter, were localized to different ER domains (Meng and Chye, 2014), the results herein using transgenic rice indicated that OsACBP4 expressed from its native promoter was targeted to the ER and PM, while OsACBP5 was directed to the apoplast of root cells. Given the waxy structure in rice tissues such as leaves, it was difficult to observe the subcellular localization of proteins in rice leaves by a fluorescence microscope (Zhang et al., 2011), hence root cells were used instead. Although rice protoplasts have been more frequently used for subcellular localization assays of rice proteins (Zhang et al., 2011; Kim et al., 2012, 2015; Zhou et al., 2018), it is not suitable for proteins that are targeted to the extracellular space and would not be applicable for testing whether a protein is delivered to the PM or cell wall. Rice root cells, that have been successfully applied for subcellular localization assays of rice protein localization (Zhang et al., 2012; Chu et al., 2018), were successfully used in this study to overcome the limitations imposed by the use of rice protoplasts. The apoplastic localization of OsACBP5 is further supported by the predicted signal peptide of OsACBP5 by iPSORT and the secretory pathway predicted by TargetP1.1 (Meng et al., 2014). It is interesting to note that the localization of OsACBP4 at the PM and ER is consistent with the results reported for its homolog, AtACBP2 in Arabidopsis (Li and Chye, 2003).

When AtACBP2:GFP fusion protein was transiently expressed from the *CaMV35S* promoter in onion epidermal cells by particle gene bombardment, signals were observed at the PM and the ER (Li and Chye, 2003). The PM localization of AtACBP2 was further confirmed by examination of confocal images of bombarded onion epidermal cells subjected to plasmolysis (Li and Chye, 2003). Furthermore, the transmembrane domain of AtACBP2 was found essential in membrane targeting because the removal of this domain impaired membrane localization (Li and Chye, 2003). Conversely, the apoplast localization for OsACBP5 was consistent with the results on the localization for its homolog in Arabidopsis, AtACBP3 (Leung et al., 2006). When AtACBP3:DsRED fusion proteins were transiently expressed in onion epidermal cells and tobacco BY-2 cells from the *CaMV35S* promoter following particle gene bombardment, signals were observed at the cell periphery (Leung et al., 2006). The extracellular localization of AtACBP3 was further verified by confocal microscopy of bombarded onion epidermal cells following plasmolysis (Leung et al., 2006). Differences in tagged-protein localization in transgenic Arabidopsis and rice imply that variation in protein subcellular localization may exist between host plants, particularly between dicots and monocots, and studies would be best investigated in the native plant. Monocot (rice) protein targeting may not be optimally processed in a transgenic dicot (Arabidopsis), perhaps arising from the different processing systems for routing between them. Likely, initial targeting to the ER in a non-native plant could not be followed up to the final destination, be it the PM or apoplast, unless it happened in the native plant.

Indeed, differences in targeting had previously been noted to occur between native and non-native plants (Leung et al., 2006; Xiao et al., 2010). For example, the homolog of OsACBP5, AtACBP3, was reported to be localized to the apoplast when a partial AtACBP3:DsRED fusion (consisting of the first 337 of 362 amino acids of AtACBP3 and lacking the acyl-CoA-binding domain) was transiently expressed in onion epidermal cells and tobacco BY-2 cells (Leung et al., 2006) in contrast to multiple signals during stable expression of AtACBP3:GFP in transgenic Arabidopsis (Xiao et al., 2010). The full-length AtACBP3:GFP when stably expressed in native Arabidopsis was targeted to the extracellular space, intracellular membranes and the ER/Golgi complex (Xiao et al., 2010). Although the results in transient expression suggested that the AtACBP3 signal sequence/transmembrane domain is deemed necessary in AtACBP3 targeting, the multiple localization of AtACBP3 in transgenic Arabidopsis indicates that other factors account for an accurate complete routing in the native plant. Some studies reported that fluorescent protein tags can affect localization results when they fused to different positions (N- or C-terminal) of a protein of interest (Snapp, 2005; Gao et al., 2012; Tanz et al., 2013). For example, GFP-ENDOMEMBRANE PROTEIN12 (EMP12) fusion was targeted to the Golgi apparatus in transgenic Arabidopsis, while EMP12-GFP fusion was localized to post-Golgi compartments and vacuoles (Tanz et al., 2013). It should be noted that GFP or DsRED was similarly fused to the C-terminal of OsACBP4 and OsACBP5 in transgenic Arabidopsis (Meng et al., 2014) and in transgenic rice in this study. Therefore, localization results obtained in this work should not be caused by the effect of differential positions of fluorescent protein tags. However, the subcellular localization of OsACBP4 and OsACBP5 when fluorescent protein tags are fused to their N-terminal remains to be determined. Taken together, these results suggest that the subcellular localization of rice ACBPs and Arabidopsis ACBPs is conserved when investigated in the respective native plant, and reinforce a need for investigations on the subcellular localization of proteins in the original organism. This study also confirmed that OsACBP4 and OsACBP5 are non-redundant proteins in plant lipid metabolism. To better understand their functions in rice, it would be interesting to investigate the effects of OsACBP4 and OsACBP5 on the composition of acyl-CoA pools, fatty acids and membrane lipids in the near future.

### OsACBP4 for Intracellular Transport of Acyl-CoA While OsACBP5 Is a Defense-Related Extracellular Protein

The subcellular localization results from this work together with the binding properties of recombinant OsACBP4 and OsACBP5 to lipids and acyl-CoA esters (Meng et al., 2011; Meng, 2012) suggest that OsACBP4 is likely involved in the intracellular transport of acyl-CoA esters and/or lipids between the PM and ER, while OsACBP5 participates in the extracellular transport of acyl-CoA esters and/or lipids. The dual localization intracellularly of an ACBP has been previously observed in mammalian cells (Hansen et al., 2008). The cellular localization of an ACBP using microinjection of fluorescently labeled bovine L-ACBP (FACI-50) in living HeLa and bovine mammary gland epithelial cells revealed its co-localization to the ER and Golgi, and the depletion of fatty acids from cells significantly reduced FACI-50 association with the Golgi, while an overload of fatty acids enhanced Golgi-association (Hansen et al., 2008), suggesting that this ACBP, proposed to be involved in vesicular trafficking, localizes to the ER and the Golgi in a ligand-dependent manner. In comparison, the relationship between the subcellular distribution of OsACBP4 with the availability of ligands needs to be explored, but such studies involving intracellular lipids would be complex given their dynamics *in planta*.

The localization of AtACBP2, the homolog of OsACBP4, to the PM and ER further supports AtACBP2 function in protection against heavy metal and oxidative stresses, as demonstrated by the overexpression of AtACBP2 or its PM-localized protein interactors (AtFP6 and AtLYSOPL2), under the regulation of the *CaMV35S* promoter in transgenic Arabidopsis (Gao et al., 2009, 2010). AtACBP2-, AtFP6-, or AtLYSOPL2-overexpressors were more tolerant to cadmium (Cd) and hydrogen peroxide (H_2_O_2_) than the WT (Gao et al., 2009, 2010). Recombinant (His)_6_-AtACBP2 was found to bind linoleoyl-CoA and linolenoyl-CoA esters which represent two precursors implicated in the repair of the phospholipid membrane and heavy metals, suggesting that AtACBP2 is involved in membrane biogenesis and protection of lipid membrane through binding to AtFP6, heavy metals and acyl-CoA esters (Gao et al., 2009, 2010). Recombinant AtACBP2 was also reported to bind lysoPC, implying that AtACBP2 also protects lipid membranes through interaction with AtLYSOPL2 and lysoPC (Gao et al., 2010; Miao et al., 2019). Furthermore, *AtACBP2pro:GUS* was expressed in embryos and guard cells in relation to *AtACBP2* function in abscisic acid (ABA) induction and drought response (Du et al., 2013a). AtACBP2-overexpressors showed enhanced drought tolerance and ABA-mediated reactive oxygen species production in the guard cells, thereby promoting stomatal closure, decreasing water loss and improving drought tolerance (Du et al., 2013a). Interestingly, *OsACBP4* has also been observed to be induced by drought treatment (Meng et al., 2014), and it remains to be tested as to whether *OsACBP4* overexpression in rice would confer drought tolerance.

The apoplast localization of OsACBP5 coincides well with its function against plant pathogens as demonstrated recently in transgenic Arabidopsis overexpressing *OsACBP5* from the *CaMV35S* promoter (Panthapulakkal Narayanan et al., 2019). OsACBP5-overexpressors showed broad-spectrum protection against plant pathogens and were more tolerant to the bacterial biotroph *P. syringae*, fungal necrotrophs (*R. solani*, *Botrytis cinereal*, *Alternaria brassicicola*) and a hemibiotroph (*Colletotrichum siamense*) than the WT and vector-transformed controls (Panthapulakkal Narayanan et al., 2019). Proteomics analysis of *R. solani*-infected Arabidopsis OsACBP5-overexpressors identified some biotic stress-related proteins including four cell wall-associated proteins, whose expression was induced after *R. solani* infection (Panthapulakkal Narayanan et al., 2019). It would be interesting to investigate if any interaction occurs between these cell wall-associated proteins and the apoplast-targeted OsACBP5 in protection of transgenic Arabidopsis. Consistently, transgenic Arabidopsis overexpressing the Arabidopsis homolog of OsACBP5, AtACBP3, had shown enhanced NPR1-dependent plant resistance against the bacterial pathogen *Pseudomonas syringae* pv *tomato* DC3000 (Xiao and Chye, 2011b). Furthermore, AtACBP3 was reported to be a phloem-mobile protein that was detected in WT Arabidopsis phloem exudates by western blot analysis and was targeted to the extracellular space as observed under immunoelectron microscopy (Hu et al., 2018). Similar to *OsACBP5* (Meng et al., 2011), *AtACBP3* expression was induced by wounding and the *atacbp3* mutant was less responsive to wounding in both local and distal leaves (Hu et al., 2018). Furthermore, GC-MS analysis showed that the levels of defense-related fatty acids such as C18:2-FA and C18:3-FA and methyl jasmonate were reduced in *atacbp3* and *AtACBP3*-RNAi in comparison to the WT (Hu et al., 2018). Subsequently, recombinant (His)_6_-AtACBP3 was identified to bind defense-related acyl-CoA esters including C18:2-CoA and C18:3-CoA esters but not methyl jasmonate (Hu et al., 2018). Taken together, AtACBP3 was destined a role in defense; it was verified mobile from shoot to root, involved in the wounding response, and influenced fatty acid levels and jasmonate content in the phloem, possibly facilitated by its binding to acyl-CoA esters (Hu et al., 2018). In comparison, the apoplast localization of OsACBP5 observed herein coincides well with a biological function in defense as demonstrated in transgenic Arabidopsis overexpressing OsACBP5 followed by proteomic analysis (Panthapulakkal Narayanan et al., 2019). These results on OsACBP5 are consistent with those on its homolog, AtACBP3 (Leung et al., 2006; Xiao et al., 2010; Xiao and Chye, 2011b; Hu et al., 2018), indicating that the Class III (Meng et al., 2011) ACBPs function in plant defense.

### OsACBP4 and OsACBP5 Do Not Appear to Translocate After Stress Treatments

No changes in the localization of OsACBP4 and OsACBP5 were noted after salt or pathogen treatments, respectively, although they were induced by salt or pathogen, respectively. These results imply that OsACBP4 and OsACBP5 do not need to translocate in the cell following stress. A plausible reason for the lack in movement may be due to a good liaison by OsACBP family members in performing their functions in lipid trafficking (e.g., apoplast localization for OsACBP5, ER, and PM localization for OsACBP4). OsACBP1 and OsACBP2 regulated from the *CaMV35S* promoter were localized to the cytosol in tobacco leaf epidermal cells and transgenic Arabidopsis cotyledon cells and plasmolyzed root cells, while OsACBP3 was targeted to the cytosol, irregular membranous, and punctate structures (Meng et al., 2014). Furthermore, OsACBP6 was detected at the peroxisomes in tobacco leaf epidermal cells, transgenic Arabidopsis cotyledon and root tip cells, as well as rice sheath cells (Meng et al., 2014). It appears that different members of the OsACBPs coordinate well to maintain acyl-CoA pools for intracellular and extracellular lipid trafficking during lipid metabolism. This possibility is feasible given the similar binding abilities of recombinant OsACBP4 and OsACBP5 to acyl-CoA esters, PCs and PAs (Meng et al., 2011; Meng, 2012). It is noteworthy that OsACBP4 and AtACBP2, both belonging to the Class II ACBPs, were each reported to be localized to both PM and ER (Figures 2, 3; Li and Chye, 2003). Indeed, TargetP1.1 predicted the transmembrane helix in OsACBP4 (amino acids 11–31) suggesting that it is associated with the ER while iPSORT predicted the presence of a putative N-terminal signal peptide in OsACBP4 (amino acids 1–30). Predotar v.1.03 further predicted OsACBP4 to be localized at the ER with a score of 0.6 (Meng et al., 2014). Furthermore, WoLF PSORT predicted OsACBP4 to be targeted at the ER, ER_PM and PM with scores of 5.5, 4.5, and 2.5, respectively^2^. Therefore, the presence of an N-terminal transmembrane helix in OsACBP4 likely directs OsACBP4 to the PM and ER to facilitate its movement between these two compartments. Similarly, Class III ACBPs (OsACBP5 and AtACBP3) are targeted to the apoplast. Given the multiple subcellular localization of AtACBP3 in transgenic AtACBP3:GFP Arabidopsis (Xiao et al., 2010), it was proposed that AtACBP3 could move from the ER to the PM before reaching the extracellular space (Xiao and Chye, 2011a). This is consistent with subcellular fractionation data that the native AtACBP3 protein and AtACBP3-GFP fusion protein were detected in the intercellular fluids, soluble and membrane fractions using anti-AtACBP3 and anti-GFP antibodies, respectively (Xiao et al., 2010). Therefore, some ACBPs do move between or amongst different subcellular compartments in the native plants. For example, OsACBP4 is likely involved in the intracellular shuttling of acyl-CoA esters and/or other lipids between the PM and ER.

Interestingly, ACBPs have been reported to influence the localization of other proteins following stress treatments. For example, under normoxia, AtACBP1 and AtACBP2 sequester the subgroup VII ethylene-responsive transcription factor RELATED TO AP2.12 (RAP2.12) at the PM through protein-protein interaction and protect it from degradation (Licausi et al., 2011). When oxygen levels were low (hypoxia), RAP2.12 dissociated from AtACBP1 and AtACBP2, was released from the PM and accumulated in the nucleus to activate hypoxia-responsive gene expression (Licausi et al., 2011). More recently, the release of RAP2.12 from the PM was found to be regulated by unsaturated long-chain acyl-CoAs such as oleoyl-CoA (18:1-CoA) and linolenoyl-CoA (18:3-CoA) (Schmidt et al., 2018; Zhou et al., 2020). These results together with the acyl-CoA binding capabilities of AtACBP1 and AtACBP2 (Li and Chye, 2004; Leung et al., 2006; Gao et al., 2009; Xue et al., 2014) imply that the accumulation of 18:3-CoA and/or 18:1-CoA esters induced by hypoxia, promotes the dissociation of RAP2.12 from AtACBP1 and AtACBP2 at the PM causing RAP2.12 entry to the nucleus to initiate downstream hypoxia signaling cascade in Arabidopsis (Licausi et al., 2011; Zhou et al., 2020). It remains to be tested whether OsACBP4 behaves like its Arabidopsis homologs, AtACBP1 and AtACBP2, in hypoxia-related stress responses and regulates the subcellular localization of other proteins.

This investigation has demonstrated the need in using a native promoter and the native plant in subcellular localization studies on a protein. Previously, AtACBP3:GFP expressed from the *CaMV35S* promoter in transgenic Arabidopsis (Xiao and Chye, 2011b) was deemed unsuitable for addressing changes in subcellular localization under different stress treatments because AtACBP3:GFP was degraded after *P. syringae* infection (Xiao and Chye, 2011b). Also, specific stress-responsive element(s) located in the native promoter may be necessary to activate gene expression after stress and may consequently affect protein localization (Duan et al., 2017). Furthermore, there have been reports that the subcellular localization of proteins may not be reliable if not expressed in the native plant (Zhang et al., 2011; Bu et al., 2014). To avoid any potential problems that may arise from use of a non-native promoter or a non-native plant, the studies reported herein employed native promoters to drive expression of the protein-tagged fusions in transgenic rice with satisfactory outcomes. Given that OsACBP5:GFP was localized to the ER in cotyledon, hypocotyl and root cells of transgenic Arabidopsis (Meng et al., 2014; Meng and Chye, 2014), we also tested whether OsACBP5:DsRed was targeted to the ER of the root cells in transgenic rice. However, no obvious ER localization was observed under normal conditions (Figure 4), indicating that OsACBP5 may not normally localize to the ER under normal conditions in rice root cells. An ER-Tracker (green) was further used to test the ER localization hypothesis for OsACBP5, signals of this ER marker appeared too strongly, and produced bleed-through artifacts in the red channel, making it difficult to conclude on whether OsACBP5 is localized to the ER membrane structures in rice, in spite of the precautions taken herein in using the native promoter in transgenic rice.

## Conclusion

In conclusion, this study addressed *OsACBP4:GFP* and *OsACBP5:DsRED* expression from their native promoters and verified their subcellular localization in transgenic rice. The results revealed that OsACBP4:GFP is targeted to the PM besides the ER, while OsACBP5:DsRED is localized at the apoplast. Furthermore, changes in the subcellular localization of OsACBP4:GFP and OsACBP5:DsRED were not detectable following salt and pathogen treatment, respectively. These results imply that OsACBP4 is likely involved in the intracellular shuttling of acyl-CoA esters between PM and ER, while OsACBP5 appears to participate in the extracellular transport of acyl-CoA esters, suggesting that they are non-redundant proteins in lipid trafficking. Furthermore, differences in tagged-protein localization in transgenic Arabidopsis and rice imply that protein subcellular localization studies are best investigated in the native plant.

## Data Availability Statement

All datasets generated for this study are included in the article/Supplementary Material.

## Author Contributions

PL and M-LC designed the research. PL, KL, S-CL, and SP performed the experiments. PL, KL, S-CL, SP, M-LC, and LJ analyzed the data. PL and M-LC wrote the manuscript. S-CL revised the manuscript. All authors have read and approved the manuscript.

## Conflict of Interest

The authors declare that the research was conducted in the absence of any commercial or financial relationships that could be construed as a potential conflict of interest.

## Footnotes

1 http://www.biorun.net

2 https://wolfpsort.hgc.jp/
