# Supplementary material for: Subcellular Localization of Rice Acyl-CoA-Binding Proteins ACBP4 and ACBP5 Supports Their Non-redundant Roles in Lipid Metabolism
Source: Front Plant Sci. 2020 Mar 24;11:331. doi: 10.3389/fpls.2020.00331 (PMC7105888; doi:10.3389/fpls.2020.00331)
Supplement: Supplementary file 1 [file Data_Sheet_1.pdf]

Table S1. Oligonucleotide primers used in this study

Gene name	Primer name	Used for	Orientation	Sequence (5’-3’)
OsACBP4 promoter	ML2465	PCR	Forward	CAGTCGACCCGTCGATCCTAGTAAGTAC (*Sal*I)
	ML2823	PCR	Reverse	CCCAAGCTTGGCCGACGCCGACGCCGA (*Hin*dIII)
OsACBP4 CDS	ML2824	PCR	Forward	CCCAAGCTTATGGGCGGCGACTGGCAG (*Hin*dIII)
GFP	ML2825	PCR	Reverse	GGGTGACCTTACTTGTACAGCTCGTCCATGCC (*BstE*II)
OsACBP5 promoter	ML2470	PCR	Forward	TAGGGATCCTGACTTCCTCACGCTCTGCT (*Bam*HI)
	ML2473	PCR	Reverse	TAGAAGCTTGCTGCACTCCTCTCTCTCTC (*Hin*dIII)
OsACBP5 CDS	ML2982	PCR	Forward	GGATCCATGGAGCTGTTCTACGAGCTG (*Bam*HI)
	ML2983	PCR	Reverse	TCTAGATTCAGCAGGGATGTCAGAACTC (*Xba*I)
DsRED	ML2984	PCR	Forward	TCTAGAATGGCCTCCTCCGAGAAC (*Xba*I)
	ML2985	PCR	Reverse	GAGCTCCTACAGGAACAGGTGGTGGC (*Sac*I)
OsACBP4 CDS	ML1060	PCR	Forward	TCGGATCCAAGGAGGACAACCTCCGAA
OsACBP5 CDS	ML1111	PCR	Forward	GAGGCTATTCCAGGATGGAT
OsACBP4	ML1109	qRT-PCR	Forward	GCATCTGGCTGCTGGTGTAG
	ML1110	qRT-PCR	Reverse	GCATTTGCATTGACAAGAATCT
OsACBP5	ML1111	qRT-PCR	Forward	GAGGCTATTCCAGGATGGAT
	ML1112	qRT-PCR	Reverse	CTGTCATGTTGGTTGATTGTAT
ACTIN	ML1115	qRT-PCR	Reverse	AGGCCGTCCTCTCTCTGTAT
	ML1116	qRT-PCR	Forward	GGGGAGAGCATATCCTTCAT

Restriction sites are underlined. CDS, coding sequence.

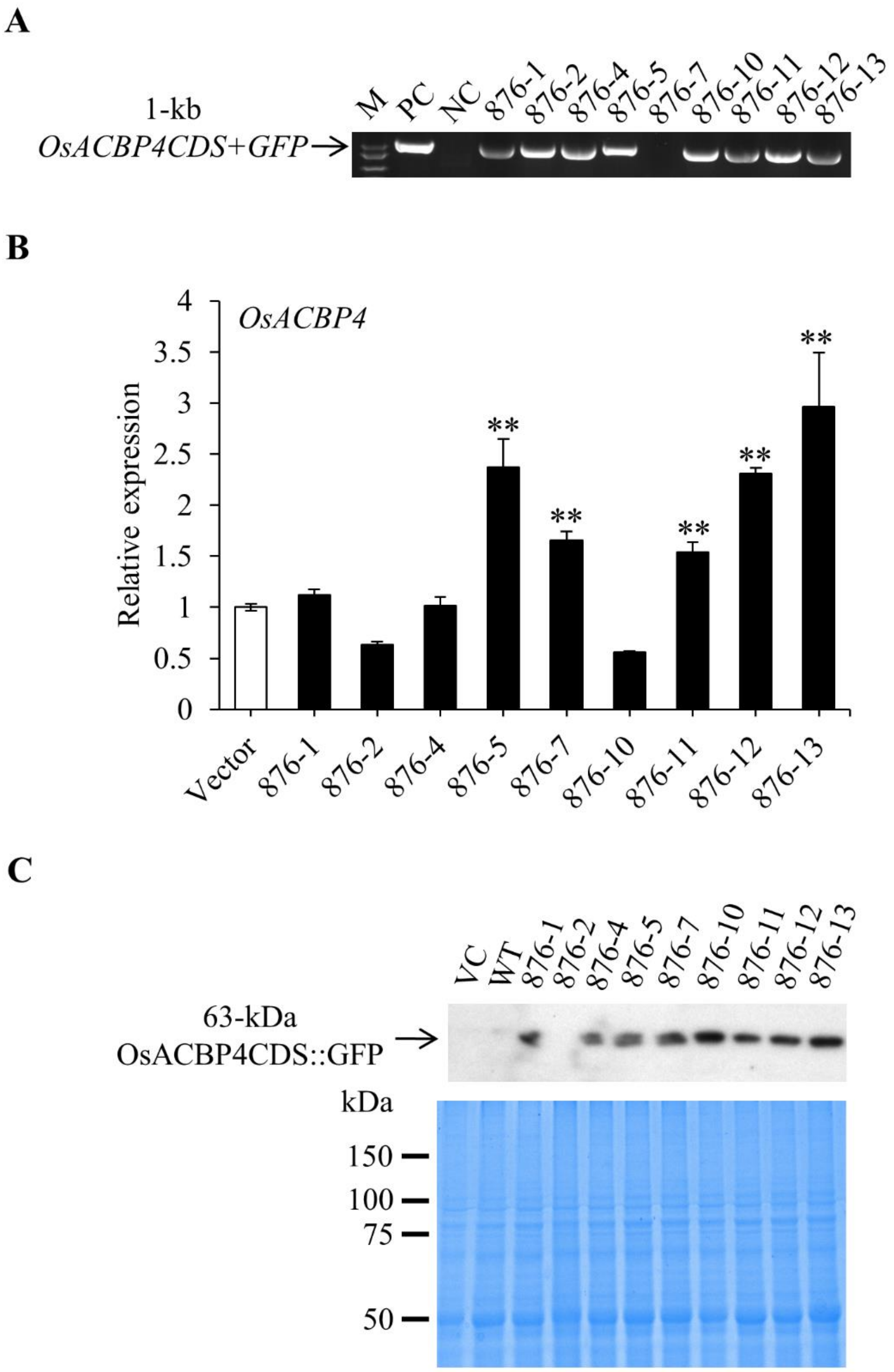

Figure S1. Characterization of representative transgenic rice expressing *OsACBP4::GFP* driven by the *OsACBP4* promoter. (A) PCR analysis on representative transgenic rice plants using *OsACBP4*-specific forward primer and *GFP*-specific reverse primer (ML1109 and ML2825). The PCR band of 1-kb *OsACBP4::GFP* is indicated. Putative transgenic *OsACBP4::GFP* rice plants are designated as 876 lines. CDS, coding sequence; M, 1 kb plus

marker; PC, positive control; NC, negative control. (B) Quantitative RT-PCR analysis on representative 21-day-old transgenic rice plants using *OsACBP4*-specific primers (ML1109 and ML1110) and rice *ACTIN* (*OsACTIN*)-specific primers (ML1115 and ML1116). Data are means ± SD of three independent replicates. Asterisks indicate a significant difference between the treatments and controls (**, $P < 0.01$ by Student's *t*-test). (C) Western blot analysis to verify the expression of OsACBP4 in representative 7-day-old rice leaves using antibodies against GFP. The cross-reacting OsACBP4::GFP band (63-kDa) is indicated by an arrow. Bottom, Coomassie Blue-stained gel of total protein (20 μg per well). VC, vector control; WT, wild type.

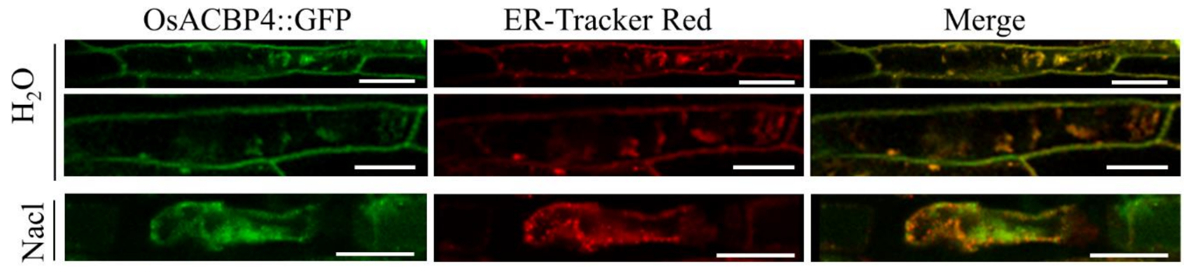

Figure S2. Subcellular localization of OsACBP4::GFP in transgenic rice after salt treatment. Root cells of 4-day-old transgenic rice seedlings were imaged by confocal laser scanning microscopy. Colocalization of OsACBP4::GFP (green) with ER-Tracker Red (red) (E34250, Invitrogen) in root cells of 4-day-old transgenic rice seedlings. Bars =20 μm. Representative images were shown after observation with consistent results from at least 20 cells.

A

B

C

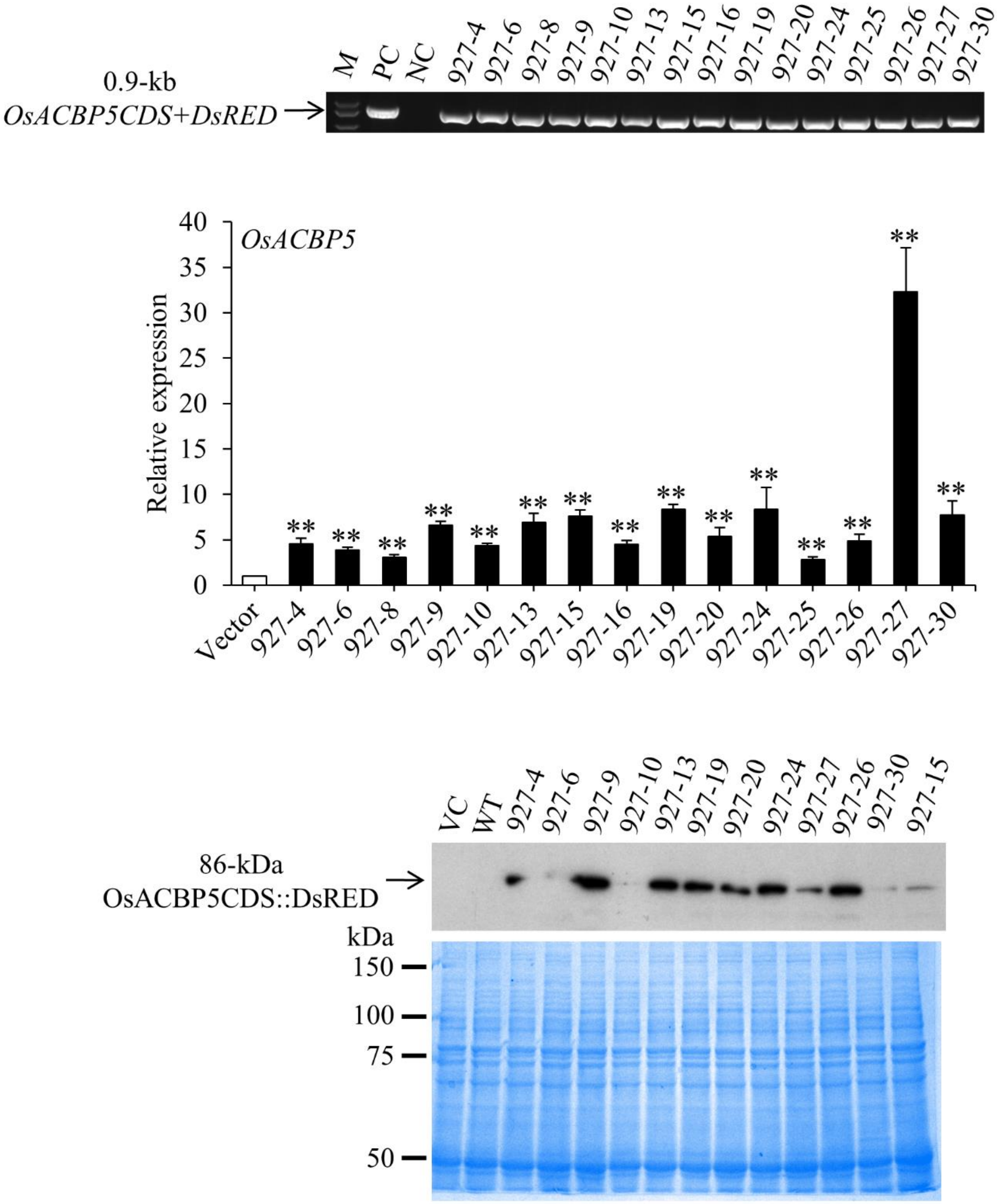

Figure S3. Characterization of representative transgenic rice expressing *OsACBP5::DsRED* driven by the *OsACBP5* promoter. (A) PCR analysis on representative transgenic rice plants using *OsACBP5*-specific forward primer and *DsRED*-specific reverse primer (ML1111 and ML2985). The PCR band of 0.9-kb *OsACBP5::DsRED* is indicated. Putative transgenic *OsACBP5::DsRED* rice plants are designated as 927 lines. CDS, coding sequence; M, 1 kb plus marker; PC, positive control; NC, negative control. (B) Quantitative RT-PCR analysis on

representative 21-day-old transgenic rice plants using *OsACBP5*-specific primers (ML1111 and ML1112) and rice *ACTIN* (*OsACTIN*)-specific primers (ML1115 and ML1116). Data are means ± SD of three independent replicates. Asterisks indicate a significant difference between the treatments and controls (**, P <0.01 by Student's t-test). (C) Western blot analysis to verify the expression of OsACBP5 in representative 7-day-old rice leaves using antibodies against RFP. The cross-reacting OsACBP5::DsRED band (86-kDa) is indicated by an arrow. Bottom, Coomassie Blue-stained gel of total protein (20 µg per well). VC, vector control; WT, wild type.
